# Mapping radon hazard areas using ^238^U measurements and geological units: a study in a high background radiation city of China

**DOI:** 10.1007/s10967-016-4717-5

**Published:** 2016-02-23

**Authors:** Hongtao Liu, Nanping Wang, Xingming Chu, Ting Li, Ling Zheng, Shouliang Yan, Shijun Li

**Affiliations:** 1 Key Laboratory of Geo-detection, Ministry of Education, China University of Geosciences (Beijing), 29 Xueyuan Road, Beijing, 100083 China; 2 School of Geophysics and Information Technology, China University of Geosciences (Beijing), 29 Xueyuan Road, Beijing, 100083 China

**Keywords:** Soil gas radon, Gamma-ray spectrometry, Radon risk mapping, Geological unit

## Abstract

In order to identify radon-prone areas and evaluate radon risk level, a soil gas radon survey combined with gamma-ray spectrometry measurements was carried out in Shenzhen City, south China. Meanwhile, the statistical analysis was applied to evaluate the distribution of measured results. This paper presents the methodology of the radon risk assessment. A radon risk map was accomplished based on a combination of soil gas radon concentration (RC), soil air permeability (Perm.) and uranium (^238^U) concentration. The results showed that the distribution of soil gas RC and radon-prone areas were closely related to geologic distribution of uranium (^238^U) and local lithology.

## Introduction

Radon (^222^Rn half-time *t*_1/2_ ≈ 3.82 days), a naturally occurring noble gas, is radioactive, colorless and odorless. It mainly originates from the ^238^U natural decay chain of rocks and soils in the earth’s crust. According to epidemiological studies, the existence of radioactive gaseous radon is considered to be the second leading cause of lung cancer after only smoking [1]. United Nations Scientific Committee on the Effects of Atomic Radiation released that ^222^Rn and its decay products accounts for 50 % of the total amount of individual background radiation dose (1.15 mSv/year per capita) [2].

There does not exist a consensus definition on a radon-prone area. Generically, ICRP defined it as an area where the radon concentration (RC) in building is likely to be higher than the national average. The objective of radon-prone area evaluations is to reduce public exposure to radon. Firstly, direct measurements of indoor radon are widely used to delineate radon-prone areas [3, 4]. Meanwhile, statistical analysis is often adopted to explore influential factors of indoor radon levels [5–11]. Secondly, gamma dose assessments based on the correlation of soil gas radon levels with ^238^U or ^226^Ra concentrations in soils and rocks have been adopted [12, 13]. Airborne gamma-ray spectrometry combined with geological information was also used to delineate radon-prone areas [14–17].

Soil gas radon, which is the predominant source of indoor radon, was regarded as a good predictor of radon potential (RP) [18–20]. Moreover, soil gas permeability (Perm.) is closely related to the migration of radon gas. Previous studies have revealed that the room-entry rate of radon increases with the rise of soil gas Perm. [3], which makes soil gas Perm. a primary criterion in radon mapping [20, 21]. RP mapping based on the measurements of soil gas radon and Perm. at a depth of 1 m beneath the ground has been accomplished in Czech Republic and Germany [22–25].

In China, although some regional surveys have attempted to delineate radon-prone areas [26], there is a lack of an accepted method of radon risk mapping. In 2013 the Radiation and Environment Laboratory at China University of Geosciences (Beijing) conducted a large scale radon survey in Shenzhen City, an area in southern China with a high radiation background. The main aim of this study is to present a detailed radon risk map based on soil gas radon, soil gas Perm. and uranium (^238^U) concentrations. A further objective is to evaluate the radon risk variation in different geological units with a spatial analysis. The accomplishment of a RP map in the study area will facilitate the radon risk assessment for human health and risk reduction.

## Materials and methods

### Study area

Shenzhen City is located on the south coast of Guangdong province, eastern Pearl River Delta Areas. It covers a total area of approximately 1953 km^2^. Shenzhen City is situated on the intersection of the west part of the north-east Lotus Hill fault and the middle part of the east–west Gaoyao–Huilai structural belt. This city is characterized by widely distributed deep faults, which would facilitate radon underground migration resulting in the increase of soil radon levels. Previous research has indicated that the average soil gas RC of Shenzhen City was 50.50 kBq/m^3^. The main lithology includes the Early-Cretaceous and the Late-Cretaceous biotite granite, Sinian metamorphic rocks, Devonian quartz sandstone and Jurassic quartz sandstone. In addition, Shenzhen City is located in the southern subtropical region with an average annual temperature of 23.0 °C and an average relative humidity of 74 %.

### Field measurements

**Fig. 1:**
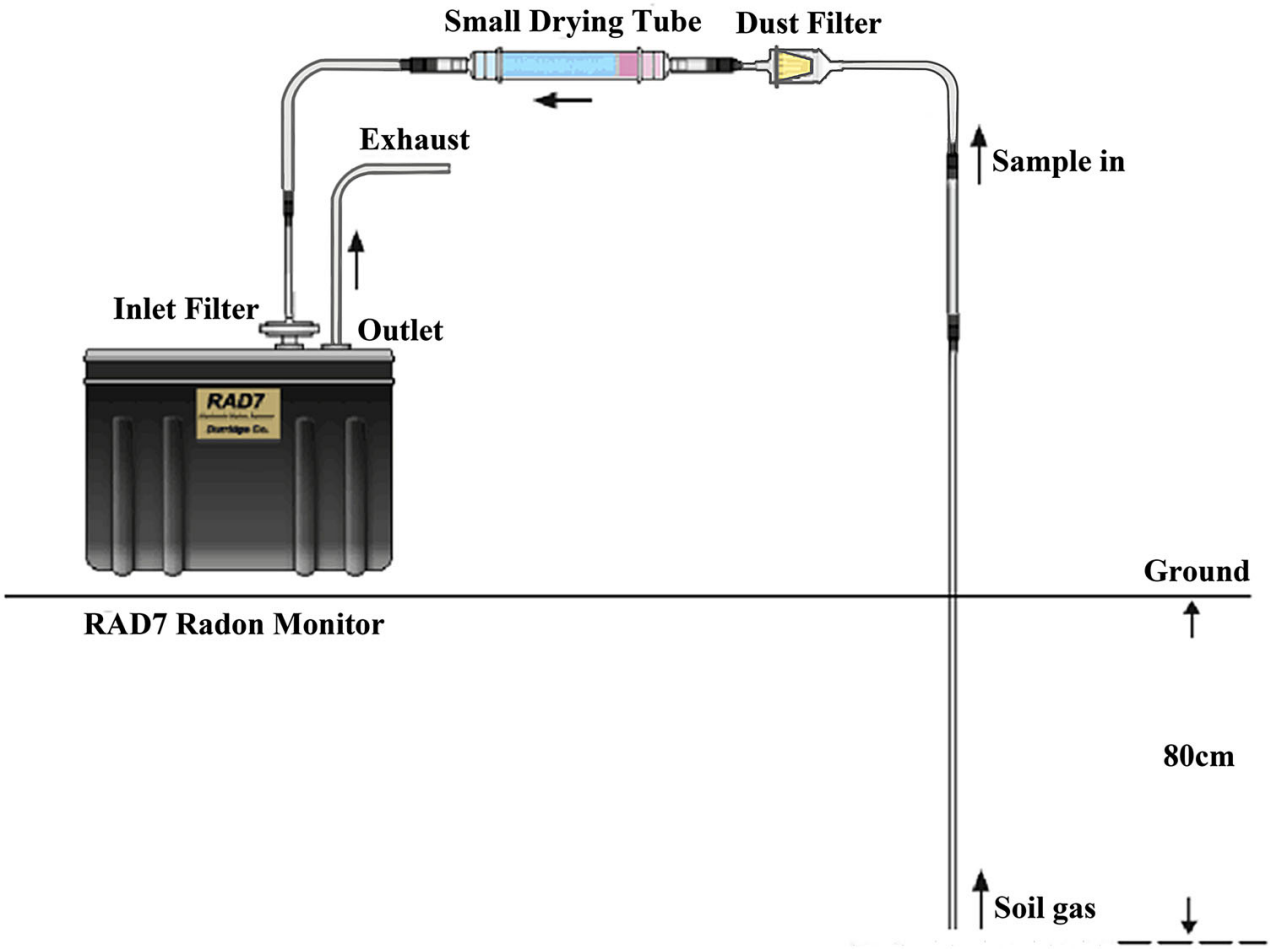
A simple schematic *diagram* of soil gas radon measurement with RAD7 equipment

**Fig. 2:**
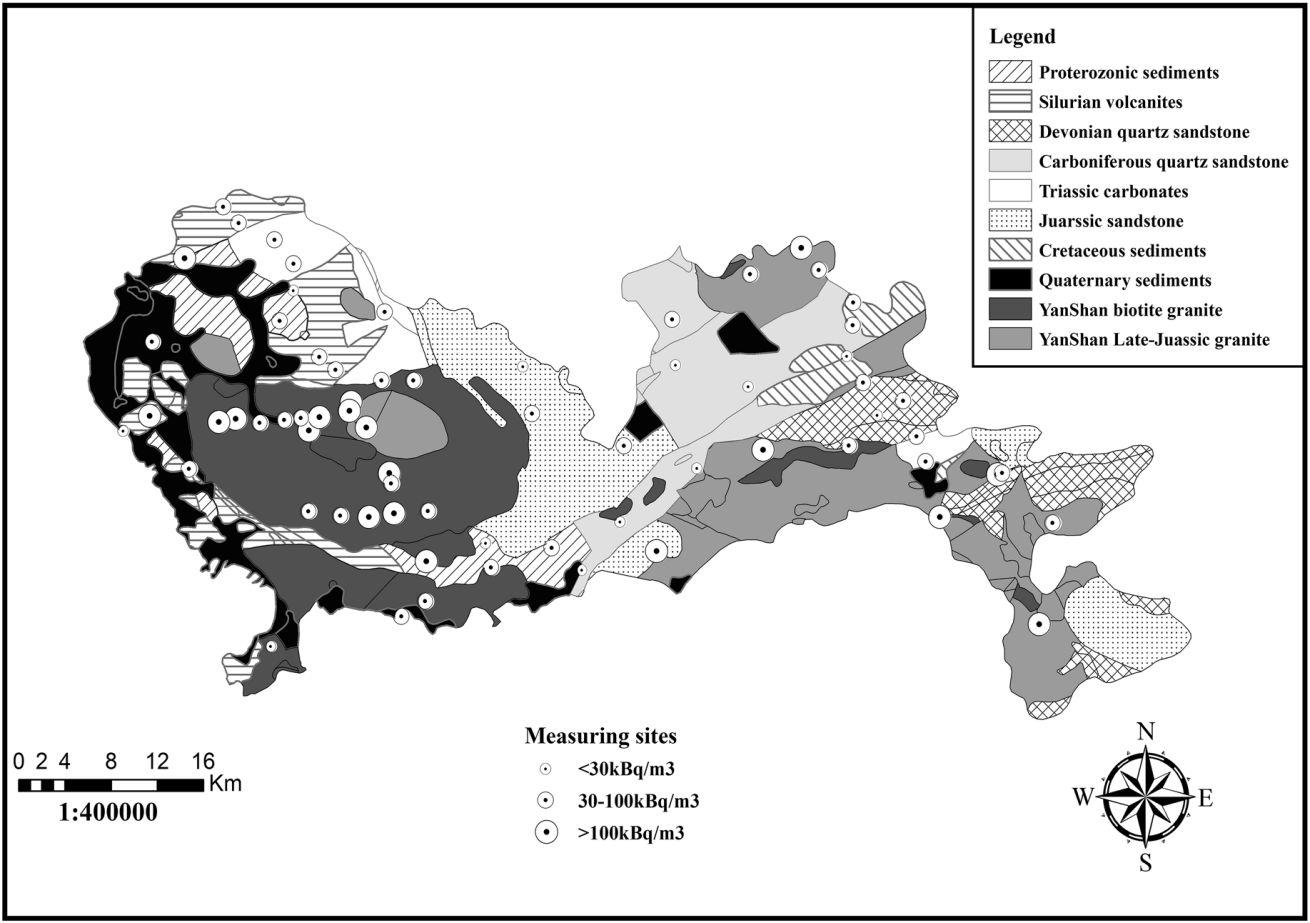
The geological sketch map of Shenzhen City with the distribution of measuring sites [size of *dot* represents the different range of soil gas radon concentration (kBq/m^3^)]

The soil gas RCs were measured by an RAD7 electronic radon detector (Durridge Co., Inc.). RAD7 mainly consists of a solid-state ion-implanted silicon semiconductor alpha detector and a 0.7 L hemispherical cavity with 2200 V potential relative to detector. The equipment was precisely calibrated at Durridge’s radon calibration facility [27], using radium (^226^Ra) source to provide controlled radon gas for calibration. According to the RAD7 user manual [28], the calibration uncertainty was 2 % (1-σ) based on counting statistics in the radon reference concentration (1.31 kBq/m^3^), and not including the uncertainty of the reference source which was evaluated to be within ±5 % (1-σ). In field measurements, a soil probe (a steel pipe with 8 mm inner diameter, 15 mm outer diameter and 110 cm length) was inserted down to a depth of 80 cm. The inlet port of RAD7 was connected to the sampling tube outlet using vinyl flexible tubes through a dust filter and a inlet filter (pore size 1 µm) which prevented dust particles and radon progeny from entering the chamber (Fig. 1). A small drying tube (CaSO_4_) was used to make sure the gas relative humidity was decreased to less than 10 %. Gas from soil interstices was pumped inside the RAD7 measuring chamber, where ^222^Rn was detected through α-decay of its daughter ^218^Po therein produced. The flow rate of pump was 1 L/min. The instrument was operated in “Sniff” mode with 3-min cycle and a single measurement at each sampling site took at least 30 min [29]. The final result was the average of stable readings in latter cycles. The equipment “Test Purge” is a necessary step before it moved to another measuring point. This survey covered an area of about 1900 km^2^ with 69 sampling sites distributed in different geological characteristics (Fig. 2). The geographic coordinates for all measurement sites were determined by a portable GPS.

Direct in situ soil gas Perm. measurements were performed before the soil gas radon measurements with Radon-Jok equipment using the same soil probe. The equipment works with air withdrawal by means of negative pressure. By using the facility, soil gas filled the packing element through the pressure difference, making the compressed packing element expand slowly. The less time the filling process took, the larger the soil gas Perm. was. The calculation of the gas Perm. was based on Darcy’s equation according to the equipment manual [30, 31].

Uranium (^238^U) concentration was measured using a portable gamma-ray spectrometer with a NaI(Tl) (Ø75 mm × 75 mm) scintillation detector (1024 channels). The energy resolution of spectrometer was 7.43 % at 662 keV. The spectrometer detector was well calibrated at China Radiometric Exploration Methodology Station of Nuclear Industry [32]. Five calibration pads were used to determine stripping ratios recommended by IAEA [33] for calculating ^238^U, ^232^Th and ^40^K activity concentrations in soils and rocks. The characteristic gamma-ray energy peaks are 1.46 MeV for ^40^K, 1.76 MeV for ^214^Bi and 2.26 MeV for ^208^Tl, respectively. Taking these three characteristic energy peaks as the positions of central peak, an inverse matrix solution spectral method was applied to acquire the conversion factors of this equipment by calculating the peak count rates of each energy range:

1 $$\left[ {\begin{array}{c} {I_{1} } \\ {I_{2} } \\ {I_{3} } \\ \end{array} } \right] = \left[ {\begin{array}{ccc} {a_{11} } & {a_{12} } & {a_{13} } \\ {a_{21} } & {a_{22} } & {a_{23} } \\ {a_{31} } & {a_{32} } & {a_{33} } \\ \end{array} } \right] \left[ {\begin{array}{c} {C_{\text{K}} } \\ {C_{\text{U}} } \\ {C_{\text{Th}} } \\ \end{array} } \right],$$

where *I*_1_, *I*_2_, and *I*_3_ are the counting rates of three energy spectrum channels (^40^K, ^214^Bi, and ^208^Tl) after deduction of background rates, in cps, *a*_11_, *a*_12_, *a*_13_,…,*a*_33_ are the conversion factors, *C*_K_, *C*_U_ and *C*_Th_ are the mass concentrations of ^40^K, ^238^U and ^232^Th, in percent for ^40^K and mg/kg for ^238^U and ^232^Th. In field measurements, the portable spectrometer was placed on the leveling and uniform ground surface, which corresponded to the calibration condition. There were no high-large buildings interfering with the measurements. Counting time is 600 s at each measuring site. The measurement procedures were in accordance with Technique Regulation of Gamma-Ray Spectrometry on the Ground issued by The Ministry of Land and Resources of China [34]. The equivalent concentrations of ^238^U and ^232^Th and the concentration of ^40^K can be acquired after getting the conversion factors of spectrometer [Eq. (1)] and calculating the peak count rates of ^214^Bi, ^208^Tl and ^40^K.

### Radon Index (RI) classification

In order to assess the radon risk of the study area, it is necessary to set up radon risk indexes. Previous research indicated a positive correlation between indoor RC and the local RI [35]. A conventional approach to quantify the RP is called the ‘Naznal RP’ [25], taking into consideration the soil gas RC and soil gas Perm. The second method to define the RP is the RI based on multivariate cross-tabulation [36]. The resulting RI is a categorical-ordinal quantity such as low, medium and high. In this study, the RI was defined based on the classification of soil gas radon, soil gas Perm. and ^238^U concentrations. Each of the three parameters was subdivided into three classes and scores were assigned to the input quantities. To be specific, soil gas radon concentration (RC for short) was classified to be 1, 2 or 3. If RC ≤30 kBq/m^3^, then grade is 1, if 30 < RC < 100 kBq/m^3^, then grade is 2, if RC ≥100 kBq/m^3^, then grade is 3 [22]. Soil gas permeability (Perm. for short) and ^238^U concentrations were also divided into three grades, respectively [13, 37] (Table 1). Finally, the result of RI for each measurement site was the sum of these three grades. The radon risk was determined following the order of high (RI = 8, 9), medium (RI = 6, 7) and low (RI = 3, 4, 5) categorization.

**Table 1:** The classification strategy of different parameters

Grades	RC (kBq/m^3^)	Permeability (m^2^)	^238^U (mg/kg)
1	≤30	1.7 × 10^−14^–4.0 × 10^−13^	2–4
2	30–100	4.0 × 10^−13^–4.0 × 10^−12^	4–8
3	≥100	>4.0 × 10^−12^	>8

## Results and discussion

### Summary statistics and regression analysis

The summary statistics of soil gas RCs, soil gas Perm. and ^238^U concentrations of all measurement sites are shown in Table 2. The results showed that soil gas RC had a minimum of 14.63 kBq/m^3^, a maximum of 369.72 kBq/m^3^, a median of 58.47 kBq/m^3^ and an arithmetic average of 85.81 kBq/m^3^ with a standard deviation of 70.94 kBq/m^3^.

**Table 2:** The summary statistics of soil gas radon concentrations (kBq/m^3^), soil gas permeability (×10^−12^ m^2^) and ^238^U concentrations (mg/kg) of measurement sites [minimum (Min), lower quartile (Q1), median, upper quartile (Q3), maximum (Max), arithmetic mean (AM), arithmetic standard deviation (SD), geometric mean (GM) and geometric standard deviation (GSD)]

Summary statistics	Counts	Min	Q1	Median	Q3	Max	AM	SD	GM	GSD
Soil gas radon concentration (kBq/m^3^)	69	14.63	39.84	58.47	105.23	369.72	85.81	70.94	87.51	2.22
Soil gas permeability (×10^−12^ m^2^)	68	0.183	1.554	4.217	9.563	25.333	6.303	5.990	1.015	5.82
^238^U concentrations (mg/kg)	70	2.019	4.030	4.878	6.973	10.295	5.482	2.023	6.847	1.61

The measurement sites in Shenzhen City were mainly distributed in four lithological units including granite, quaternary, sandstone and backfill. The Kruskal–Wallis test showed that there was a statistically significant difference in the soil gas RCs (*χ*^2^ = 30.81, *p* = 0.000 < 0.05) and ^238^U concentrations (*χ*^2^ = 29.39, *p* = 0.000 < 0.05) among different lithological units (Table 3).

**Table 3:** Different values [arithmetic mean (AM), median and interval] of soil gas radon concentrations (kBq/m^3^), logarithm values of soil gas permeability and ^238^U concentrations (mg/kg) of measurement sites in different lithological units

Lithology	Counts	Soil radon concentration (kBq/m^3^)			${\text{Log}}_{10}^{{({\text{soil}}\;{\text{gas}}\;{\text{permeability}})}}$			^238^U concentrations (mg/kg)		
		AM	Median	Interval	AM	Median	Interval	AM	Median	Interval
Granite	42	115.50	95.38	27.84–369.72	−11.463	−11.411	−12.737 to −10.596	6.43	6.23	3.67–10.29
Quaternary	7	27.81	25.68	16.33–41.84	−11.417	−11.086	−12.732 to −10.746	4.03	4.19	2.02–5.00
Sandstone	15	45.42	41.76	14.63–105.03	−11.366	−11.334	−12.682 to −10.696	3.58	3.31	2.29–6.39
Backfill	3	58.45	62.44	45.85–67.07	−11.054	−10.806	−11.637 to −10.719	5.73	5.61	4.55–7.01

**Fig. 3:**
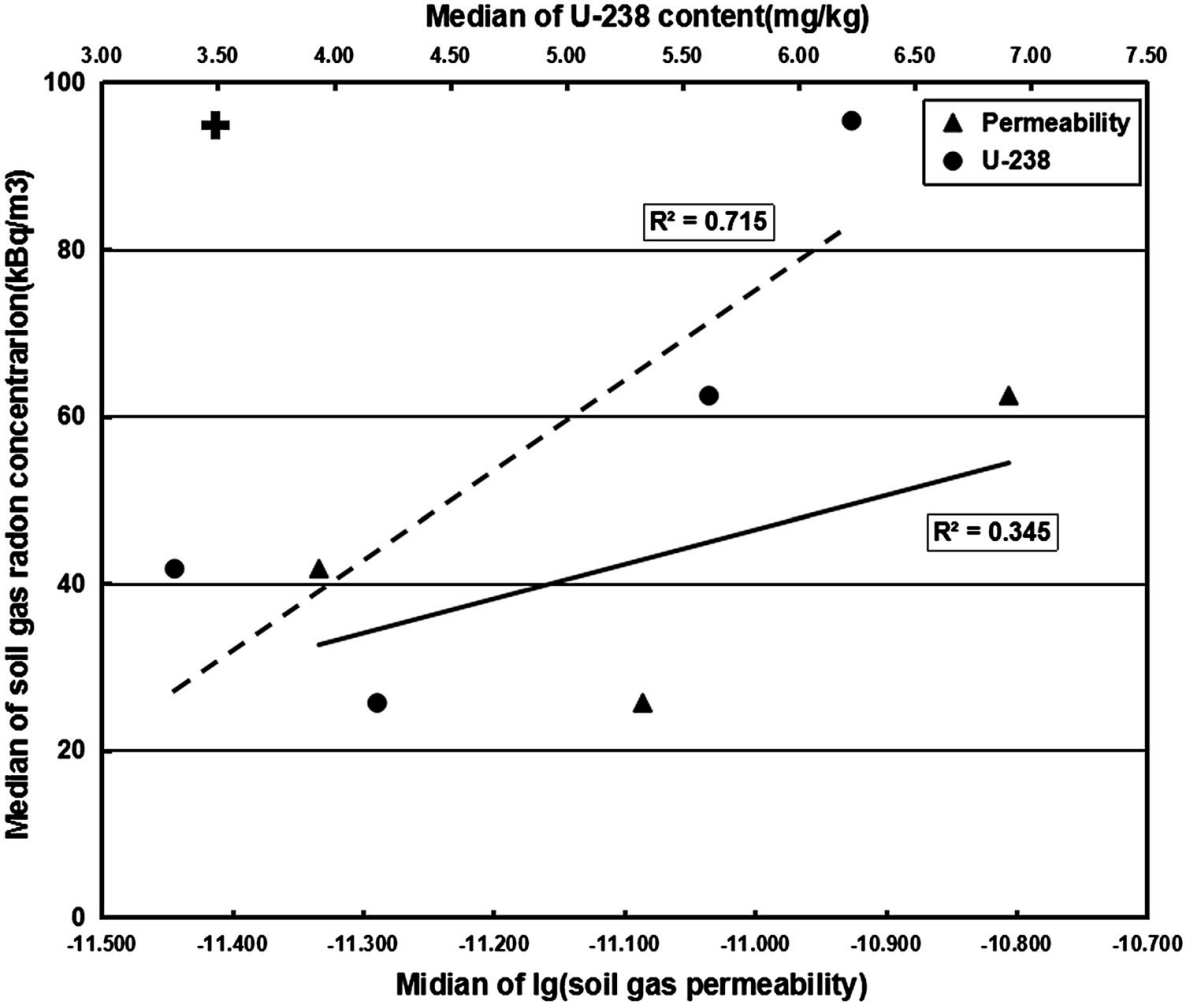
Regression analysis between measurement results (*solid line* and *triangle*: a weak correlation between medians of soil gas radon concentrations and soil gas permeability (the *cross symbols* representing the outlier value in granite areas), *dashed line* and *solid dot*: a positive linear correlation between medians of soil gas radon concentrations and ^238^U concentrations)

Medians of soil gas RC and soil gas Perm. (logarithmic scale) from four main lithological units were conducted a simple bivariate regression analysis, which showed a weak significant linear relationship (*R*^2^ = 0.345) even if the outlier (the values in granite areas) was not considered (Fig. 3). However, the relationship between the soil gas RCs and ^238^U concentrations revealed a high positive linear correlation (*R*^2^ = 0.715; Fig. 3), which could also be proved by the Pearson’s correlation analysis that included all measurement sites (*r* = 0.709, *p* = 0.000 < 0.05) [38].

### Radon risk assessment within study area

**Fig. 4:**
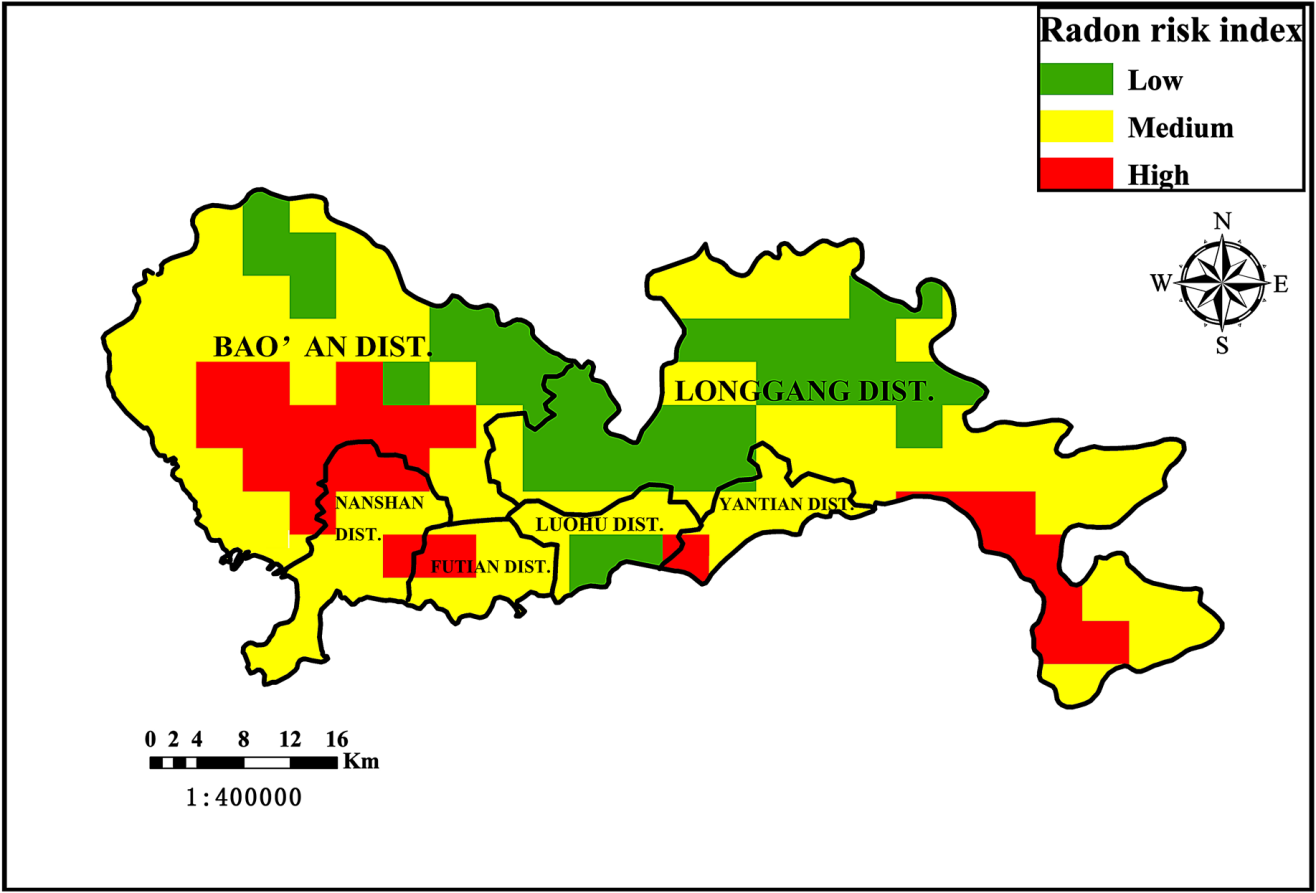
The radon potential map of Shenzhen City (1:400,000)

As an overall result, soil gas RCs were higher in the western and southern parts of Shenzhen City (Fig. 2), especially in the Midwest areas. The regionalization of the radon risk map in Shenzhen City was realized by means of a grid-based and distance-weighted interpolation procedure using Software ArcGis10.2 (Fig. 4). Each grid element represented an area of 4 km × 4 km. For each raster element without measuring site, the three nearest measurement points in the same geological unit were allocated [39].

This study provided a general distribution of the radon risk on a large scale. High radon risk areas were widely distributed in the western part of Shenzhen City. Additionally, the radon risk evaluation based on the administrative areas showed that the central part of the Bao’an District, the northwest part of the Nanshan and Futian Districts were radon-prone areas in the study area. The distribution characteristics of the RP in Shenzhen City were closely related to the local lithology. The highest radon risk areas concentrated in the mid-western areas, where Yenshanian granite was widely distributed.

## Conclusions

Soil gas radon distribution and radon risk assessment could be observed intuitively through the RP map of Shenzhen City. The research showed that:

1. The arithmetic average of soil gas RC in Shenzhen City was 85.81 kBq/m^3^, which was 12 times higher than that of the other 144 cities in China (7.3 kBq/m^3^).
2. Statistical analysis indicated that there were significant differences in the concentrations of soil gas radon and uranium (^238^U) among the different lithological units. There was a close correlation between radon-prone areas and geologic distribution of uranium (^238^U).
3. This survey demonstrated that the Nanshan District and the Bao’an District, which were covered with a large range of Yanshanian period granite rocks, were typical high radon risk areas.

The application of this methodology and the study of radon mapping in China is currently in progress. Further radon surveys may be extrapolated to a bigger range in China.
